# Big boned: How fat storage and other adaptations influenced large theropod foraging ecology

**DOI:** 10.1371/journal.pone.0290459

**Published:** 2023-11-01

**Authors:** Cameron C. Pahl, Luis A. Ruedas

**Affiliations:** Department of Biology and Museum of Vertebrate Biology, Science Research and Teaching Center—246, Portland State University, Portland, Oregon, United States of America; State Museum of Natural History, GERMANY

## Abstract

Dinosaur foraging ecology has been the subject of scientific interest for decades, yet much of what we understand about it remains hypothetical. We wrote an agent-based model (ABM) to simulate meat energy sources present in dinosaur environments, including carcasses of giant sauropods, along with living, huntable prey. Theropod dinosaurs modeled in this environment (specifically allosauroids, and more particularly, *Allosaurus* Marsh, 1877) were instantiated with heritable traits favorable to either hunting success or scavenging success. If hunter phenotypes were more reproductively successful, their traits were propagated into the population through their offspring, resulting in predator specialists. If selective pressure favored scavenger phenotypes, the population would evolve to acquire most of their calories from carrion. Data generated from this model strongly suggest that theropods in sauropod-dominated systems evolved to detect carcasses, consume and store large quantities of fat, and dominate carcass sites. Broadly speaking, selective forces did not favor predatory adaptations, because sauropod carrion resource pools, as we modeled them, were too profitable for prey-based resource pools to be significant. This is the first research to test selective pressure patterns in dinosaurs, and the first to estimate theropod mass based on metabolic constraints.

## Introduction

Although agent-based models (ABMs) are used extensively in ecology, and continue to generate important, novel theories [1], few have been written to test hypotheses in paleontology. In an ABM, experiments are based on “Agents”, which correspond to a virtual representations of modelled object entities. These agents interact within a virtual world along with other autonomous agents—of their kind or of distinct kinds—and in doing so, generate data about the systems they are meant to imitate [1, 2]. We used evidence from a previous ABM to propose that the supply of sauropod carrion generated by background mortality rates would have been sufficient for carnosaurs such as *Allosaurus* to have sustainably met their energy budgets if they were ecological analogues of modern vultures [3]. Sauropods were as large as many modern cetaceans [4, 5], and their carcasses therefore densely rich with calories. We hypothesized that the existence of these large dinosaurs created an overabundance of meat resources, which relaxed selective pressure on predatory adaptations in many theropod taxa, resulting in carrion specialist dinosaurs, rather than obligate predators. The results of our analysis also suggested that the high proportion of theropods in some fossil assemblages could be explained by foraging ecology dynamics shaped by overabundance of sauropod carrion, and challenged consensus views as to the manner by which large theropods acquired most of their calories.

The hypothesis outlined above follows logically from existing established data [3], but Agents (i.e., virtual subject animals) in the model we wrote to test the hypothesis had no concept of differential reproductive success based either on phenotype heredity or individual variation. Because of this, we were not able to measure how natural selection could have favored the evolution of adaptations in theropod species. To test this latter question, we wrote a new ABM that allowed a simulated allosaur population to evolve over time. It again was generally based on fauna of the Morrison Formation, but meant to loosely represent almost any sauropod system in the Mesozoic Era. The ABM included as its primary active components Allosaur Agents (meant to approximately simulate *Allosaurus*), Prey Agents (meant to approximately simulate live prey, based loosely on *Stegosaurus*), and sauropod Carcass Objects. Allosaur and Prey Agent behaviors were informed by those modeled in Wolf-Sheep ABMs [6], while sauropod Carcass Objects were derived from those modeled in our previous research [3]. Importantly, the new model incorporated a system of heredity into its Allosaur Agents, whereby phenotypic traits could be passed from parents to offspring within a framework of variable, semi-random constraints. Half of these traits influenced predation success of individual Allosaur Agents against Prey Agents, while the other half influenced scavenging success on Carcass Objects. While most analyses infer dinosaur ecology based on morphological traits analogous to those in modern taxa, this model allowed us to analyze experimental data about how theropods evolved in response to resource opportunities in the environment. In other words, this ABM represents the first attempt to measure selective pressure or fitness potential in dinosaurs. We also were able to estimate an energetically optimum body mass for these animals, an important goal of the metabolic theory of ecology [7, 8] that has never been attempted for dinosaurs.

## Methods

### Experimental design

The simulated environment was a flat, toroidal, 70 x 70 km grid, meant to simulate a featureless 4,900 km^2^ landscape. Agents were of two types: Allosaur Agents and Prey Agents. Agents shared the landscape with stationary, ephemeral, randomly generated sauropod Carcass Objects, which decayed over time. The model functioned within a framework system of heredity, such that Allosaur Agents would evolve toward the phenotypic attributes that contributed the most to their fitness, which we measured based on reproductive potential of each individual Agent [9]. We compared adaptive traits against lifetime reproductive success, and calculated the probability of reproduction based on each phenotype. Data were collected at runtime; code for setup and analysis can be found at: https://github.com/cmrn-crmns-phl/big_boned. We divided the model into 365-day cycles to approximately match the duration of a single year, and collected data over a span of 279 years.

#### Allosaur agents

Allosaur Agents were derived from Wolf agents in Wolf-Sheep models; as such, they were not programmed to hunt in packs, establish territories, or migrate. They were modeled as endothermic *Allosaurus* individuals, pursuant to recent research suggesting these animals exhibited high aerobic capacities comparable to those of mammals and birds [10]. Agents were initialized with resource needs commensurate with those of a 2,000 kg carnivore, based on the field metabolic rate (FMR) equation for birds, yielding energy demands of ca. 29 kg of meat per day [11, 12]. Importantly, Agents were written with dynamic metabolic budgets, so their individual resource needs were recalculated continually based on body mass, a preferred modelling approach in this kind of ABM, and allowed us to estimate adaptive optimum body mass [7]. This means that while all Allosaur Agents began their lives needing 29 kg of meat per day to survive, an individual that reached 2,500 kg in mass required 33 kg, and a 4,000 kg animal required 46 kg. Likewise, smaller Allosaur Agents were more physiologically economical, and at 1,900 kg, needed 27.7 kg of meat per day. If an Allosaur Agent’s energy dropped below 1,840 kg, or 92% of its original mass, it died of starvation. Large vertebrates usually can survive several weeks without food, and sometimes emerge from long periods of resource deprivation having much less than 92% of their healthy mass; we thus considered this to be a very conservative starvation limit [13, 14].

Allosaur Agents moved about the space randomly unless they located nearby Prey Agents or Carcass Objects, which were targeted as feeding opportunities. Allosaur Agents were able to move 3.0 km per day. Our previous model [3] used a traveling limit of 1.0 km per day in its Allosaur Agents, which we posit was overly conservative, given that most large animals consistently travel much further than this in one day. Polar bears, for example, are considered inefficient terrestrial walkers, but nonetheless are nomadic, and usually travel between 1.0 and 5.0 km per hour, covering thousands of kilometers in a year; elephants have a slightly higher walking speed and cover greater distances [15, 16]. *Allosaurus* themselves likely were able to reach a top speed of ca. 21 km per hour [17], which makes our model’s daily travel limit both energetically realistic and scientifically cautious.

In addition, the new ABM allowed endothermic Allosaur Agents to survive with lower carcass detection ranges than detailed in our previous work, where results indicated that endotherms were unable to survive with detection ranges below 9 km [3]. To simplify the model, Allosaur Agents reproduced asexually, and the population produced offspring at a rate of 0.02. Offspring inherited phenotypes from their parents within the hereditary constraints we designed for this model.

The model began with 12 Allosaur agents, and was limited to a maximum of 30 at any given time. Although our previous analysis suggested that carrion from sauropods may have supported hundreds, or even thousands, of *Allosaurus*-sized theropods in an area of the size tested here (4,900 km^2^), and fossil evidence suggests these animals likely were that common [10], we set the maximum at 30 because it is both conservative and consistent with known population densities of modern endothermic hypercarnivores [3, 18].

#### Phenotypes, advantage factors, and expression

Allosaur Agents were assigned six heritable phenotypic attributes; each attribute had an advantage factor that modified it along a gradient that influenced the success of the organism (Table 1). Three of the attributes modified agent success in ways commonly associated with vultures and other scavengers. These attributes were “carcass detection_range”, “tailfat”, and “dominance” [19–21]. The other three attributes: “bite_force”, “hearing”, and “binocular_vision”, influenced Allosaur Agents’ success during predation attempts on Prey Agents due to their importance in predation success [22, 23]. Five different expressions determined the individual’s advantage factor for a given trait: “extra_small”, “small”, “medium”, “big”, and “extra_big” (Table 2).

**Table 1:** Phenotype influence on Allosaur Agents.

Phenotype	Ability
detection_range	Distance in km an Allosaur Agent potentially could detect a Carcass Object
tailfat	Amount of meat in kg an Allosaur Agent could consume and store as tail fat, per feeding event, per day
dominance	Allosaur Agent feeding priority at a Carcass Object site
bite_force	Increased or decreased odds of killing target by Allosaur Agent during encounter with Prey Agent
hearing	This attribute slowed a Prey Agent target if detected by an Allosaur Agent; Allosaur Agents with a high advantage factor slowed Prey Agents more than did an Allosaur Agent with a low advantage factor
binocular_vision	Distance in km an Allosaur Agent could detect a Prey agent using vision only; this attribute was fundamentally the same as detection_range, but applied only to Prey Agents.

**Table 2:** Phenotype expressions and advantage factors.

Phenotype expression	advantage factor	tailfat	carcass detection range	binocular vision/prey detection range
extra_small	0.3–0.65	11.4–24.7 kg	1.5–3.25 km	0.15–0.325 km
small	0.65–0.9	24.7–34.2 kg	3.25–4.5 km	0.325–0.45 km
medium	0.9–1.1	34.2–41.8 kg	4.5–5.5 km	0.45–0.55 km
big	1.1–1.35	41.8–51.3 kg	5.5–6.75 km	0.55–0.675 km
extra_big	1.35–1.7	51.3–64.6 kg	6.75–8.5 km	0.675–0.85 km

For example, consider an Allosaur Agent freshly initialized at the first step of the model. Expression of its carcass detection range was randomly assigned to “small”, which in turn set its detection range to a random value between 3.25 and 4.5 km. If instead it had been set to “medium”, its carcass detection range would have been between 4.5 and 5.5 km. All zero-generation Allosaur Agents had phenotypic attributes set randomly. Attributes were not subject to nominal evolutionary tradeoff penalties [24] such that in a mechanical sense, Allosaur Agents were able to evolve both predatory and scavenging adaptations if the selection environment were to favor both. Importantly, this hereditary framework allowed the system to mimic genetic phenomena of individual variation, penetrance, and expressivity [25, 26].

Although phenotypes were named based on adaptations known to be favorable either to hunting or scavenging, the attributes themselves were not always meant to replicate natural abilities. Some were proxies, meant to characterize any traits that upon which a selective pressure could act to favor ecological specialization in any direction and to any degree. For example, Allosaur Agents had a “hearing” attribute, but were not programmed with a concept of hearing or sound. In life, predators typically experience strong selection on their auditory sense, and rely on it to approach prey stealthily, particularly in ambush situations [27]. To illustrate this type of advantage in our ABM, which was soundless, the “hearing” attribute determined how quickly a Prey Agent was able to move if detected by an Allosaur Agent. Greater expression of the phenotype (e.g., better “hearing”) slowed a Prey Agent down more as it moved about the space, which allowed the Allosaur Agent to approach it more closely, and increased the likelihood of a terminal collision between the Allosaur Agent and its Prey Agent target. In this way, “hearing” allowed Allosaur Agents to engage in a coarse, imprecise version of ambush behavior, although the behavior itself technically was applied without any cognitive concept of hunting on the part of the Allosaur or Prey Agents.

Another proxy phenotype represented fat storage in Allosaur Agents, modeled as character “tailfat”. Fat storage is a critical and ubiquitous component of animal energetics. Indeed, fat may comprise more than half of an animal’s total mass [28–30]. Selective pressure on fat storage and hyperphagy may be of special importance to egg-laying animals [31] and animals that survive in environments of periodic resource deprivation [32, 33], as did many theropod species, including *Allosaurus*. Theropod tails are thought to have counterbalanced the anterior mass of their bodies, or to anchor the *M*. *caudofemoralis* during locomotion [34], but other important tail functions have not been explored comprehensively; we propose that fat storage was one of these functions. It stands to reason that large dinosaurs stored fat in their tails and fat-body organs just as do modern reptiles [35], particularly so because their tails potentially constituted such large energy reservoirs: theropod tails could have comprised 1/6^th^ of the animal’s total mass [34]. For these reasons, and although Allosaur Agents were not modelled with physical mouths, “tailfat” fundamentally determined the volume of meat an agent could consume and store in a single day if it encountered a feeding opportunity (Table 2). It is likely that large theropods evolved hyperphagy, hibernation, aestivation, and other common adaptations to overcome environmental changes in resource abundance; we did not simulate those attributes in the current model because they would have made the survival threshold too permissive for meaningful selective pressure to occur.

In the case of Carcass Object detection ranges, all “medium” agents had between 4.5 and 5.5 km of olfaction range, which we considered a realistic baseline for these animals. Olfactory prowess largely is determined by the volume of the olfactory bulb [36, 37], rather than its relative size. Humans have unremarkable olfaction abilities but can reportedly smell sauropod-sized whale carcasses from 6 km [38] with an olfactory bulb volume of only 40 mm^3^ (0.04 ml) [37]. Smaller mammals, such as domestic dogs, have olfaction centers of 0.18 ± 0.02 ml in volume and can detect carrion from multiple kilometers [39, 40]. Coyotes can reportedly detect carrion from more than 20 km, and arctic foxes are able to detect carrion from potentially 60 km [39]. The olfactory processes in *Allosaurus* comprised almost 25% of the length of its brain and may have occupied up to 15,000 mm^3^ (15 ml) of volume [41–43]. It therefore would be unexpected if *Allosaurus*, with an enlarged olfactory apparatus up to 375 times the olfactory volume of a human, 83 times that of a domestic dog, and consequent significant dependence upon its sense of smell [41, 44], were unable to detect rotting sauropod-fall carcasses from similar distances.

Attributes such as “dominance”, which had no measurable physical components, used the advantage factor as the phenotype expression. An Allosaur Agent with “medium dominance” of 1.0, was able to displace an agent with a -0.23 “small dominance” individual if they both targeted the same feeding site (Table 2). Although large individual body size itself often confers dominance in life, we separated this trait from agent mass because other attributes often contribute to dominance as well. These attributes can include behavioral adaptations, such as aggression in wolverines for example, which are not preserved in the fossil record, but consistently win resources for animals [21]. They also might include fleshy caruncles and display features as seen in today’s vultures, but that are unlikely to fossilize.

Similarly, “bite_force” influenced an Allosaur Agent’s odds of making a kill by modifying a baseline probability of 0.1, but was not a modeled indicator of jaw strength because Allosaur Agents did not have physical mouths. Rather, bite force was one of a series of characters involved in biting and ingestion, and was meant to represent any adaptation that changed an Allosaur Agent’s probability of predation success, whether from jaw strength, venom, or other specializations. We set the baseline for this attribute at 0.1 because large prey specialists such as lions and tigers can have predation success rates between 0.15 and 0.2 [45]. Lions and tigers evolved to specialize as hunters of large prey, so we consider their success rates to be higher than they would be in an unspecialized condition. By setting the predation success baseline at 0.1, the population of Allosaur Agents had room to evolve toward a more optimum predatory fitness if competition for resources were to favor it.

The attribute for “binocular vision” was fundamentally the same as carcass detection in that it defined the spatial envelope by which Allosaur Agents could detect Prey Agents. The value itself was 1/10 of the corresponding carcass detection_range values, because predators typically detect and engage prey targets from foreshortened, local proximities. Binocular vision evolves to improve visual targeting for grasping and biting behaviors, and prey targets are typically selected and approached from within 150 m [46] so this attribute was meant as a broad proxy for prey detection, rather than a realistic one.

#### Reproduction and heredity

**Fig 1:**
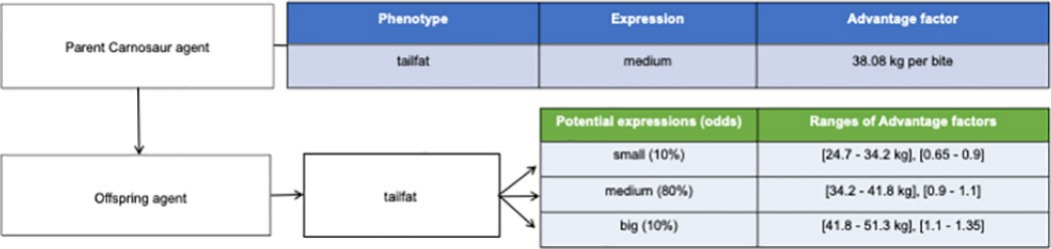
Phenotype heredity. Allosaur Agent offspring phenotypes were determined based on a hereditary gradient of Advantage Factors, which were bound to an expression magnitude. In this example, the parent Agent will have offspring with an 80% chance of having “medium” tailfat, but 10% chance to be small, and 10% to be big.

When an agent reproduced, it could only do so within hereditary constraints governed by its phenotype expressions (Table 3). For example, an agent with “medium” carcass detection_range had a 10% chance of producing “small” or “big” carcass detection offspring, and an 80% chance of producing a “medium” detection_range offspring (Fig 1). It also meant that a “medium” carcass detection animal could never produce offspring with 8 km carcass detection_range, and a “large” carcass detection animal could never produce offspring with 2 km of detection_range. It took a minimum of 4 generations for an “extra_small” phenotype agent to antecede an agent with an “extra_big” phenotype, which limited the potential for generational leapfrogging.

**Table 3:** Phenotypes and potential offspring phenotypes.

Parent phenotype expression	Potential offspring expression
extra_small	extra_small, small, small
small	extra_small, small, medium
medium	small, medium, big
big	medium, big, extra_big
extra_big	big, big, extra_big

#### Prey Agents

Prey Agents represented 2,000 kg herbivores, loosely based on the mass of *Stegosaurus* [47], which likely were in a size class that allowed them to be targeted by predators the size of *Allosaurus* [48], whereas sauropods in contrast were too large to constitute prey. *Stegosaurus* were typical of Prey Agents modeled in other ABMs [6]: they moved about the space randomly, but were not required to eat, did not migrate, did not retaliate against their attackers, or overcome any non-predatory survival stressors. Although it is accepted that *Stegosaurus* and other armored dinosaurs were able to use defensive weaponry in life, we did not incorporate these attributes into Prey Agents because they would have introduced a survival cost on Allosaur Agents. We did not want such a cost to interfere with the conservative goals of our hypothesis testing, but the dangerous nature of dinosaurs such as *Stegosaurus* almost certainly made them less desirable as resource opportunities for predators of all types. They were able to travel 3 km per day and had no athletic advantage against Allosaur Agents. Their speed did not change except when they were slowed by the “hearing” attribute of a pursuing Allosaur Agent. Prey Agents were allowed no unauthorized breeding; rather, the population was maintained to at least 20 Prey Agents for the duration of runtime. If a Prey Agent was killed, it became a 2,000 kg Carcass Object and could attract Allosaur Agents that detected it, while other Prey Agents were triggered to reproduce and return the population to its minimum. In this way, they functioned as a hypothetical all-female population of cloned dinosaurs, which—like some West African frogs—could spontaneously change sex when confined to a single-sex environment [49, 50]. This guaranteed that at any given time, the number of predation opportunities on the modelled landscape was 5 times greater than the number of scavenging opportunities. These rules also protected Prey Agents from extinction if it were the case that Allosaur Agents evolved to exploit them as their primary resource. It should be noted, however, that while ornithischians such as *Stegosaurus* were less common and less diverse in the Morrison Formation than were sauropods [51], their population densities are not known and could have been different from those we modelled.

### Carcass Objects

Carcass Objects had a mass of 10,000–25,000 kg, except in the case of Prey Agents that were killed, which became 2,000 kg Carcass Objects. Sauropod Carcass Objects appeared randomly on the landscape, decayed over time, and lost mass as Allosaur Agents consumed them. Carcass Objects disappeared when they reached 20% of their original mass, to account for bones and other low-value elements of the cadaver, although it should be noted that even these elements of modern carcasses often are consumed rapidly by terrestrial scavengers [52]. The total number of sauropod Carcass Objects was limited to 5 at any given time, which we consider conservative, given that bone beds often preserve the remains of at least this many sauropod individuals from single mortality events. Evidence suggests these dry season death assemblages were targeted by large theropods at all levels of decomposition [53, 54]. In life, carcasses of dinosaurs probably were distributed according to well-understood factors of large herbivore life histories, such as along annual migration routes, and during seasons of nourishment stress, and would not have been difficult for theropods to locate. To mimic these effects on carcass production levels, Carcass Objects only were generated during the calendar year 1^st^ quarter, the 3^rd^ quarter, and the final 45 days of the 4^th^ quarter, which limited the total number of sauropod Carcass Objects to 15–28 per year. Although our goal was not to estimate true population densities, these numbers probably are below the expected adult mortality from a population density of only 0.33 sauropods per square km, which is much lower than other estimates in the literature [55–58]. As such, we consider these parameters to be conservative enough that they probably do not reflect true values in life.

Each Carcass Object had a 0.02 chance of randomly being removed from the occurrence environment at any point in time, to ensure they were available unpredictably. This variable is not realistic because sauropod carcasses were enormous and by mass alone would have been able to remain on the landscape for months or longer: potentially up to six years [3, 59]. Without random carcass removal, carcass persistence was governed only by decay and consumption; thus, it often was possible for Allosaur Agents to survive and reproduce even without significant phenotypic advantages over their peers. An Allosaur Agent with “small tailfat” and “medium dominance” could remain on a Carcass Object for weeks, reach its maximum profitable mass, and flood the population with offspring of similarly benign attributes during that time. Random removal of Carcass objects also helped to prevent particularly dominant Allosaur Agent from drowning out other phenotypes. Otherwise, they would have been capable of monopolizing resource islands, and easily outcompeted other phenotypes. Accordingly, interruption of some Carcass Object feeding events by semi-random removal reduced, but did not fully correct these problems, because even a 10,000 kg sauropod Carcass Object had enough calories in gross to support a 4,000 kg Allosaur Agent for more than 150 days if bones were not consumed, i.e., nearly half a year’s worth of its foraging needs.

We consider our Carcass Object mass values, and the number of Carcass Objects we modeled, to be conservative. Evidence suggests that large sauropods were common and their species widespread, and this probably is not due to sampling biases or preservational distortion [51, 60]. It is difficult to imagine how an ecosystem defined by so many species of whale-sized herbivores could somehow produce low amounts of carrion, either in absolute numbers or proportionally to other resources. For example, it would have been possible to model an occurrence environment with populations of just 2 coexisting species of *Apatosaurus*, both of which reached or exceeded 20 tonnes in adulthood. Natural populations do not indefinitely increase or decrease, so both species’ populations would need to be large enough, with enough adults, to be reproductively stable [7, 61] for at least tens of thousands of years. With sufficient computational resources, it would be possible to recreate their hypothetical life histories, including egg-laying, mating seasons, demographic structures, migrations, food requirements, natural mortality, and even herding behaviors [62, 63]. But even a detailed, 2-species model of this type would ignore populations of up to 9 other species of 10–50 tonne diplodocids found contemporaneously in the same environment [60, 64]. These also would require their own reproductively sustainable populations, and necessarily would have experienced annual mortality favorable to large carcass specialists because a certain number of individuals die annually in all vertebrate populations. And beyond diplodocids, populations of species in the several other local sauropod families also necessarily contributed carrion from natural attrition every year. Long-lived, prolific egg-layers, like sea turtles and presumably sauropods as well, can experience annual adult mortality up to 16% [61]. Thus, all other factors aside, attempts to model sauropod carrion abundance conservatively, or to assume their carcasses appeared rarely, are themselves difficult to justify. Even if population crashes temporarily rendered a few sauropod populations adultless, or sunk recruitment levels below sustainable levels during catastrophes, the population crashes themselves would have inundated the environment with dead sauropod calories. It is very possible that certain types of environmental crises were beneficial to theropod survival because of the resultant influx of megaherbivore carrion. Be that as it may, we hypothesize that the true amount of biomass in sauropod-dominated ecosystems either has been neglected entirely, or grossly underestimated in all dinosaur research, including our own [3].

## Code availability

Data were generated at runtime; code to recreate this experiment can be found at: https://github.com/cmrn-crmns-phl/big_boned.

## Results

### Selective pressure and evolutionary trends

**Fig 2:**
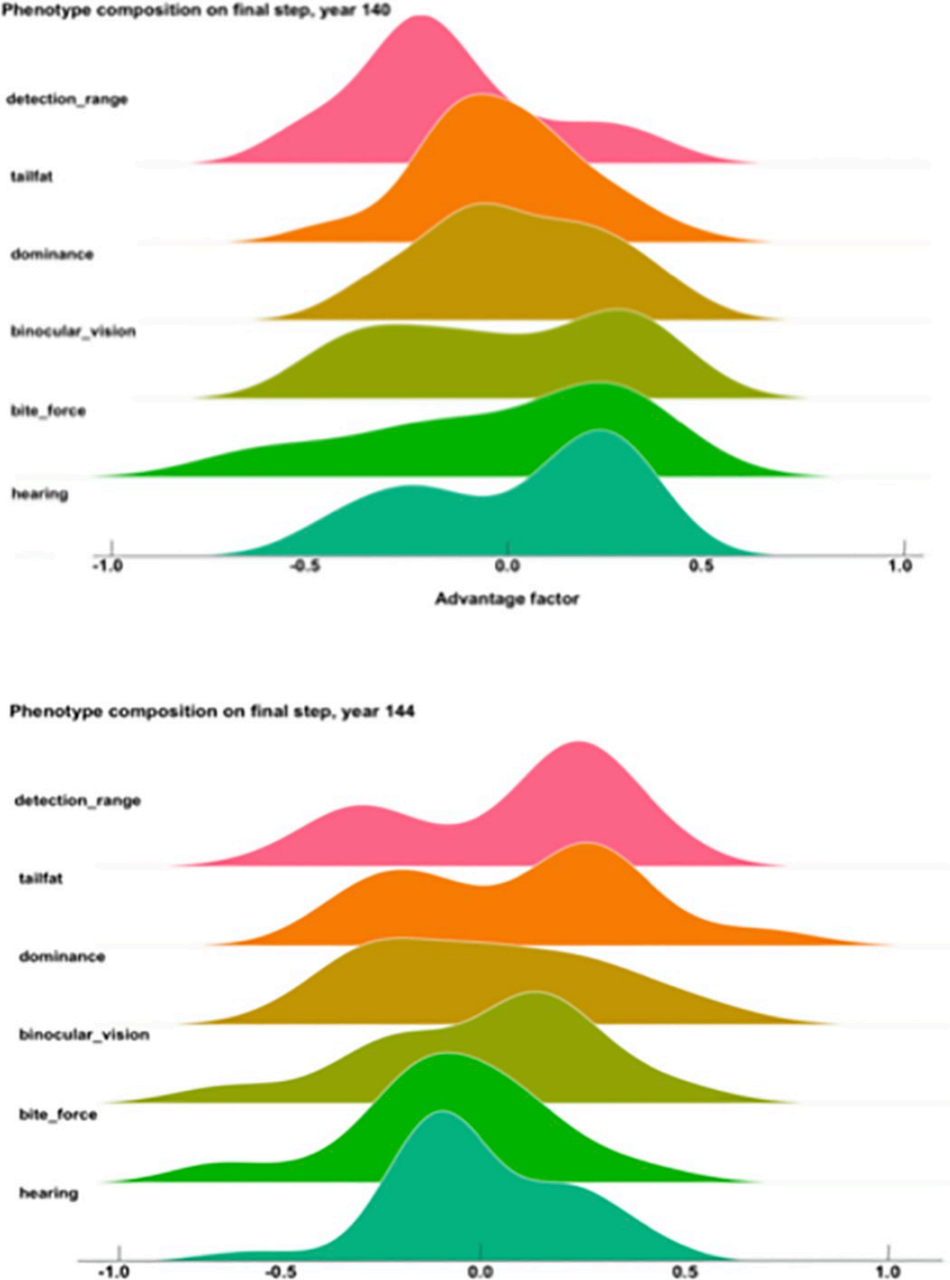
Phenotype compositions. In year 140, most surviving Allosaur Agents on the final step had improved hearing and bite_force attributes compared to the founding population, but detection_range and tailfat were negatively selected. Year 144 resulted in a population with improved detection_range, while hearing and bite_force in contrast were negatively selected.

**Fig 3:**
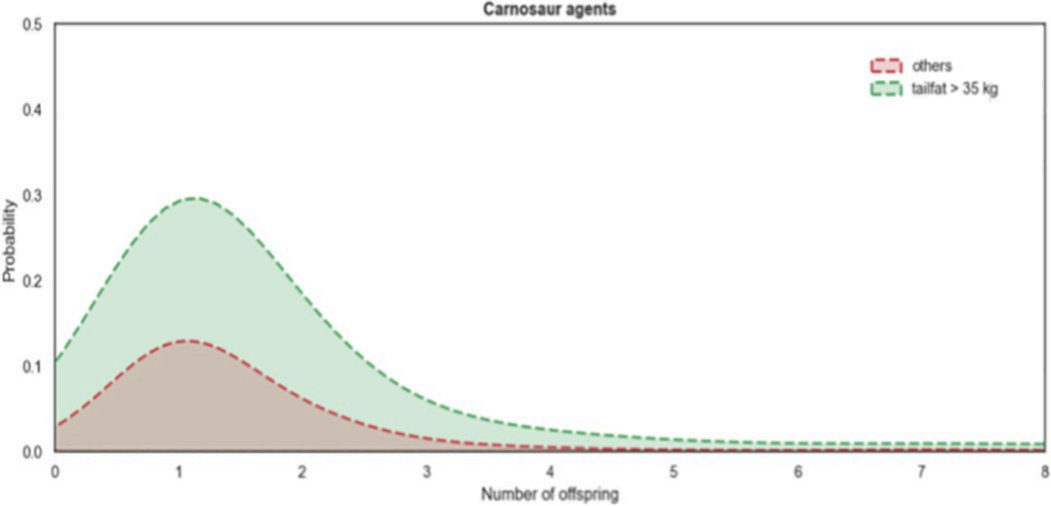
Reproductive probability distribution of high tailfat individuals. Kernel density estimation for the lifetime reproductive success distribution of Allosaur Agents. Allosaur Agents with tailfat above 35 kg per feeding event had more than 0.28 likelihood probability of producing 1 offspring during their lifetimes, whereas in all others, the probability was ca. 0.12, i.e., a factor of 2.33 times higher for tailfat > 35kg than for all other tailfat phenotypes. This fitness pattern agrees with other empirical and modeled population data, regardless of life-history scheme [66].

**Fig 4:**
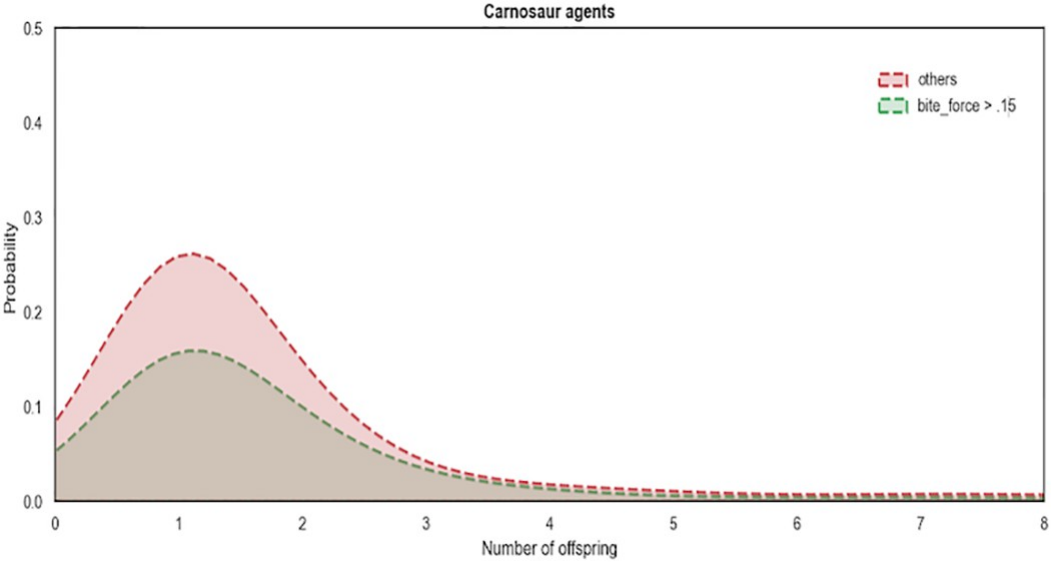
Reproductive probability distribution of high bite_force individuals. Reproductive success distribution of high bite_force individuals. Agents with high bite_force were less likely to produce offspring than all others.

**Fig 5:**
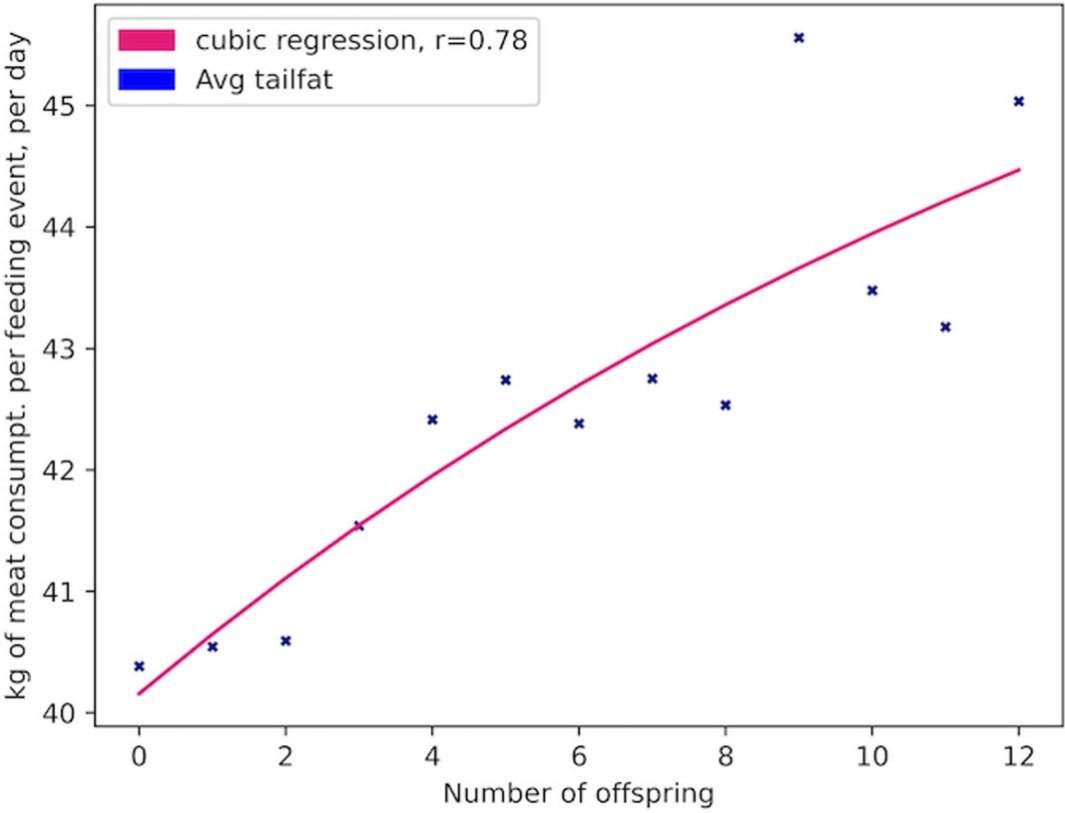
Reproductive success of Allosaur Agents *vs*. tailfat expression. Reproductive success of Allosaur Agents vs tailfat expression.

**Fig 6:**
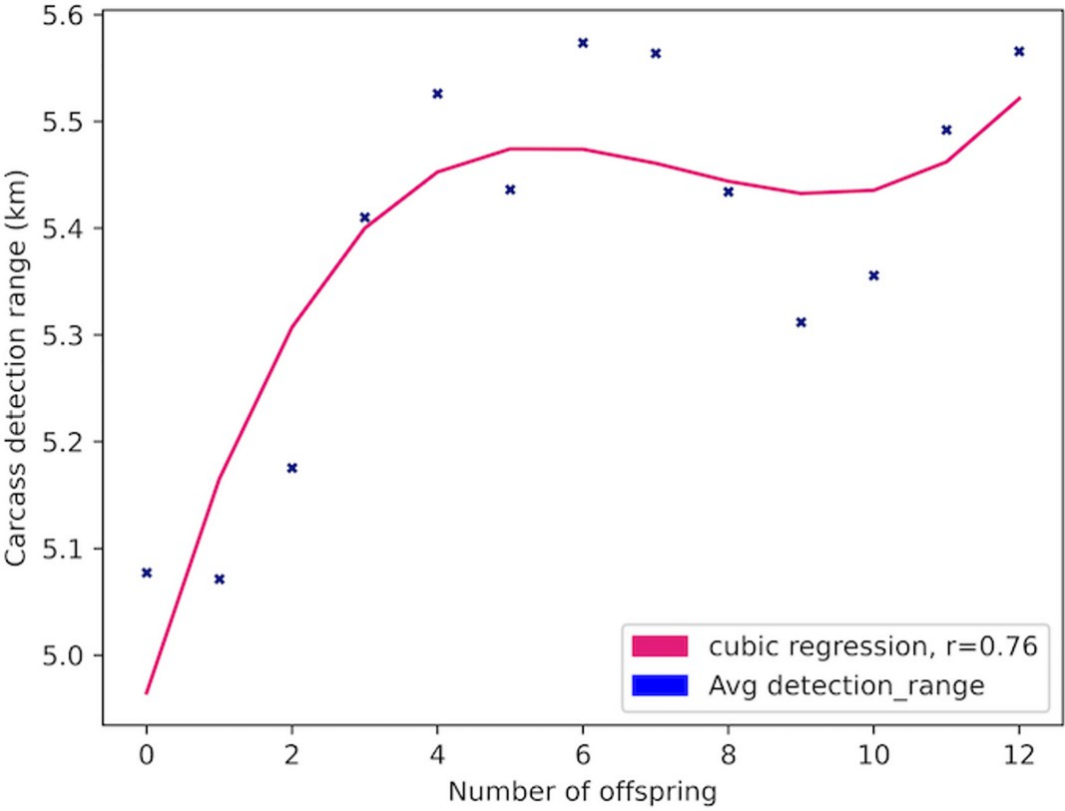
Reproductive success of Allosaur Agents vs carcass detection_range. Average Allosaur Agent carcass detection_range *vs*. lifetime reproductive success. Carcass Object detection_range was consistently higher in the most successful Allosaur Agents, demonstrating a clear selective advantage for theropods that could locate sauropod carrion better than their peers. These data suggest it therefore is likely that animals like *Allosaurus* used olfaction to detect food opportunities in a similar manner.

**Fig 7:**
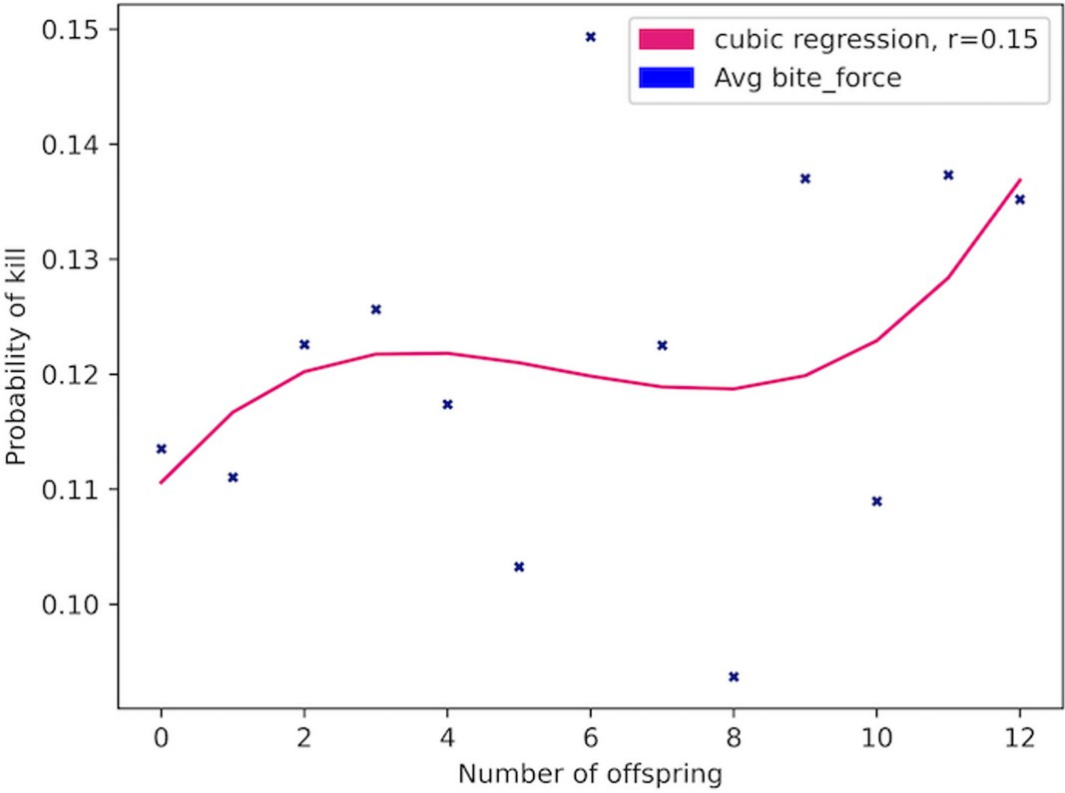
Reproductive success of Allosaur Agents *vs*. bite_force. The bite_force attribute determined the likelihood an Allosaur Agent would successfully kill a Prey Agent upon encounter. Selective pressure did not favor this attribute in our model, and given that allosaurs and most large allosauroids were poorly adapted to overpower prey, we think it is likely that the mechanisms at work in this model were broadly similar to those present in Mesozoic ecosystems.

Over the course of 279 iterations (equivalent to years) of the model, 23,092 Allosaur Agents were produced, an average of ca. 83 per year; 58% of these perished rapidly without reproducing, while the remainder produced between 1 and 12 offspring during their lifetimes. Only 37 Allosaur agents were able to produce 12 offspring, so we consider these and other high output animals to represent ultra-high fitness individuals in the environment. This pattern provides encouraging support for the structure of our model, because it mimics empirical observations in modern species, such as the Orinoco Crocodile (*Crocodylus intermedius*; Reptilia: Crocodilia: Crocodylidae), and Eastern Kingbird (*Tyrannus tyrannus*; Aves: Passeriformes: Tyrannidae). In both cases, a large share of the population are reproductively unsuccessful in a given year, while a small number of higher-fitness animals are responsible for the majority of progeny [65, 66]. Scavengers, predators, and generalists evolved as dominant foragers in different years, so in one sense, we could hypothesize that the foraging environment was ambiguously favorable both to predators and scavengers (Fig 2). However, hypothetically, inferior phenotypes can be stochastically dominant in populations, particularly during short intervals; we therefore measured fitness based on overall reproductive potential [67–69]. Scavenger phenotypes had higher reproductive fitness than non-scavenger phenotypes (Figs 3–6; Table 4). For example, Allosaur Agents with tailfat above 35 kg, had a 0.28 probability to produce 1 offspring, while all other agents had an approximate probability of 0.12; i.e., Allosaur Agents with > 35 kg tailfat were 2.33 times more likely to produce offspring (Figs 3 and 5; Table 4). Conversely, agents with high bite_force were 0.46 times as likely to produce offspring as all others (Figs 4 and 7; Table 4). It is possible that negative selection for this attribute was caused by the time commitment and uncertain success odds involved in predation. High bite_force individuals were more likely to kill Prey Agents, which were less profitable than free carrion; specialization on this resource pool therefore deprived Allosaur Agents of more consistently lucrative feeding opportunities, whereas scavenging phenotypes were able to profit from dead Prey Agent carcasses as well as sauropod Carcass Objects. Binocular_vision, which governed detection of Prey agents, also was selectively advantageous when above the baseline 0.5 km. This result is interesting because in life, *Allosaurus* had laterally directed eyes and therefore were not visually adapted for macropredatory activities [3, 70]. It is possible that stegosaurs and other appropriately-sized prey animals in *Allosaurus*’ environment were less common than we modeled them or, more likely, that sauropod carrion production was much higher. In either case, we hypothesize that selective pressure on visual targeting systems would have been weak or nonexistent in real conditions, and the fossil record reflects this strongly because *Allosaurus* and its relatives had poor vision compared to most predators [70].

**Table 4:** Mean attribute expressions grouped by reproductive success.

Offspring produced	Population bin of agents	dominance	detection_range km	Tailfat: meat kg/consumption day	bite_force	binocular_vision	hearing	mass kg	energy_budget kg meat/day
0	13440	0.02	5.08	40.38	0.11	0.51	0.02	1850	27.23
1	6413	0.02	5.07	40.54	0.11	0.51	0.02	1856	27.3
2	1702	0.03	5.18	40.59	0.12	0.51	0.03	1896	27.69
3	481	0.03	5.41	41.54	0.13	0.52	-0.04	2005	28.77
4	269	0.06	5.53	42.42	0.12	0.53	-0.07	2162	30.28
5	182	0.02	5.44	42.74	0.1	0.52	0.02	2233	30.96
6	126	0.06	5.57	42.38	0.15	0.53	0	2207	30.71
7	122	0.03	5.56	42.75	0.12	0.52	0.02	2847	36.54
8	92	0.02	5.43	42.53	0.09	0.51	0.08	2668	34.96
9	65	0.08	5.31	45.56	0.14	0.54	0.02	3107	38.79
10	54	0	5.36	43.48	0.11	0.53	-0.01	2988	37.76
11	42	0.07	5.49	43.18	0.14	0.54	0.05	2422	32.72
12	37	0.08	5.57	45.04	0.14	0.55	-0.04	2243	31.05

### Energetically optimum body mass

Recent models indicate that a species’ body mass reaches a stable plateau when the conflict between feeding efficiency and thermoregulatory costs approaches a balance point [7, 8, 71, 72]. In our ABM, reproductively successful Allosaur Agents averaged 2,345 kg. Agents of this size required ca. 33 kg of meat per day, above the initialized need of 29 kg, which makes sense because agents evolved to have high tailfat consumption abilities, which averaged 42.5 kg of meat per consumption day. We hypothesize that this range of values could signal an adaptive body mass plateau if *Allosaurus* were an endotherm, and it may be corroborative that our body mass results generally agree with other estimates for adult *Allosaurus* [17].

Reproductively successful Allosaur Agents sometimes exceeded 4,000 kg, but agents of this size comprised less than 1% of the total agents created. These animals of necessity had “extra_big tailfat” required to accommodate their > 50 kg daily meat budgets and often produced multiple offspring (Table 5). This result also helps corroborate some observations in the literature. Several authors have inferred that uncommonly large, tyrannosaur-sized theropods inhabited the Morrison, based on denticle sizes in bite marks on bone surfaces, and fragmentary skeletal material [59]. In our model, large theropods appear to have succeeded in part based on luck. They were able to use 2–3 carcasses near one another from an early point in the simulation, which increased their total consumption opportunities. However, not all “extra_big tailfat” Allosaur Agents located multiple sauropod carcasses immediately, and not all Allosaur Agents that did so had tailfat attributes large enough to support such high metabolic costs.

**Table 5:** Extremely large Allosaur Agents.

energy_budget	tailfat	offspring	mass kg	hearing	bite_force	dominance	detection_range	binocular_vision
57.19	62.37	9	5,492	0.22	0.21	0.16	6.09	0.39
57.52	59.55	8	5,539	-0.31	0.22	-0.15	3.87	0.44
48.25	63.52	5	4,280	-0.01	0.19	-0.07	5.93	0.43
48.19	64.43	9	4,273	0.04	0.15	0.27	6.31	0.38
48.23	50.87	7	4,279	0.32	0.17	0.27	7.01	0.34

## Discussion

### Theropod scavenging research

Questions about scavenging efficiency in dinosaurs have been considered by others, and some have employed similar methods as our own to answer them. A computational modeling project about scavenging efficiency in *Tyrannosaurus rex* concluded that it would have been difficult for *T*. *rex* to survive on carrion alone if exposed to conditions as observed in the modern Serengeti [73]. Although our previous research also concluded that *T*. *rex* probably was a predator [3], critical assumptions in [73] may influence its immediate relevance to the present study for three primary reasons. First, we think the authors of [73] modeled theropod carcass detection range too restrictively. Second, population density in their model was not high enough to represent dinosaur ecosystems, particularly those with sauropods. Third, their work was principally about *T*. *rex*, which did not coexist with sauropods except at the extreme southern tip of its known distribution. That paper’s conclusions therefore only are remotely applicable to the present research.

The *T*. *rex* agents in the primary model of [73] had a carcass detection range of only 80 meters. We found this value too low to be meaningful because *T*. *rex* is known to have had very well–developed senses of olfaction and vision [41, 74]. Large theropods such as *Allosaurus* almost certainly were able to detect carcasses of giant sauropods from multiple kilometers away, because although their vision was poor, their brains were dedicated largely to olfaction [41, 43]. As mentioned previously, even humans are able to detect decaying carcass odors from up to 6 km away [38]; it would be unreasonable to assume that *T*. *rex*, or indeed any theropod, would have been inferior to humans in this respect, and certainly not by such a vast margin. We think this alone would be reason enough to question the results obtained by [73], particularly if used to explain foraging ecology of theropods in dissimilar ecosystems. And even if smaller theropod species were able to leverage their lower metabolic costs as an advantage in carrion searching, as suggested, they likely would have been outcompeted by larger theropods in other ways. Dominance hierarchies routinely determine success in carcass-site contests, and smaller animals often lose, even when they arrive early [21]. It also is very possible that the display features preserved in carnosaurian skull ornamentation evolved in part to facilitate dominance while scavenging. It is interesting that selective pressure was strong enough to favor horns and rugosities in the skulls of these animals repeatedly over the course of their natural history, but not strong enough to favor predatory mainstays such as bite force or stereopsis. In any case, large aromatic sauropod carcasses probably were detectable from distances much greater than 80 meters. Carcass detection range predicted scavenger survival and fitness in our previous research [3]. A population of ectothermic theropods were sustained by sauropod carrion with detection range of only 3 km, even when they could travel no more than 1 km per day. Mesothermic and endothermic agents required 6 km and 9 km of detection range for population-level support on this resource under the same conditions, and it is reasonable to expect that most large theropods met these adaptation criteria easily [3].

The authors of [73] also modeled dinosaur abundance based on population densities in modern mammalian biomes, where smaller animals comprise a greater share of the overall community than large-bodied animals. Because of this, they estimated 5 tonne tyrannosaurs to constitute only 0.1% of the population, and large herbivores to be similarly rare. This assumption appears to be unsupported by fossil evidence, because *T*. *rex* lived in an environment dominated by large-bodied animals. *Triceratops*, at 5–10 tonnes, was by far the most abundant species, sometimes numerically comprising approximately 40% of the total fauna in the Hell Creek Formation, varying from 28% in the Lower Hell Creek to 69% in the Upper Hell Creek [75], while [73] estimated their representation as 0.4% of the mass of the community. The data from [75] instead suggest that—depending on the estimate of mass one uses for each taxon—*Triceratops* would have constituted approximately 53% of the total mass of Hell Creek Formation dinosaurs, or over one order of magnitude greater than the estimate used by [73]. *Tyrannosaurus rex* made up over 25% of the fauna in some strata—it too was very common—and for the summed Hell Creek fauna constituted 24% numerically, and ca. 26% by mass, again dependent on the mass estimate applied. In other places, such as the Morrison Formation, much larger sauropods were speciose, and overwhelmingly more abundant than smaller-bodied animals like ornithischians, and published data are clear that this representation is not due to preservational biases [60, 64]. It therefore is unlikely that the model in [73] represents dinosaur communities, even approximately, given the population density assumptions they relied on.

In addition, the model of scavenging opportunities proposed by [73] probably was not applicable to most Mesozoic ecosystems, particularly those with large sauropods. Those authors [73] modeled carrion supply based on estimates derived from the modern Serengeti, but the Serengeti has no sauropod-sized animals, so at best we consider it an inappropriate comparator. Our research about scavenging dynamics is concerned principally with sauropod carrion as a resource, and how this resource uniquely influenced the evolution of large theropods. The Morrison Formation is a much better foundational system than the Serengeti for our questions, because it was dominated by multiple species of sauropod above 20 tonnes in adult mass (11 of 43 taxa [60, 76]), and was much more productive than any modern biome [77]. While [73] probably are correct that *T*. *rex* rarely encountered sauropod carcasses, it is difficult for us to agree that a large theropod in the Morrison Formation would experience such a challenge, because sauropods were ubiquitous in that faunal system. And because most sauropod systems were characterized by harsh seasonal changes in productivity, it is logical that natural mortality of large sauropods would have occurred annually, probably during the dry season. This would have provided an abundance of low-risk, high-reward resources for local carnivores of any type, probably for weeks or months. Bone beds containing multiple sauropod individuals from annual mortality events, including drought-induced ones, are fairly common in the Morrison Formation, and evidence that theropods targeted them at all stages of decomposition fits perfectly with our hypothesis [53, 54]. Our research [3] suggests that carnosaurs such as *Allosaurus* most likely evolved to capitalize on sauropod carrion generated from these recurrent mortality events, and that evolutionary pressures favored scavenging for them very strongly because of this. Researchers would not be likely to reach this conclusion by modeling a system without sauropod carrion, whether it was the Serengeti, or another biome.

Other empirical evidence supports our hypothesis, because it has been demonstrated that specimens of *Allosaurus* were able to survive even when they were physically disabled, and incapable of hunting [78, 79]. Given what we know, it is likely these individuals met their energy budgets by scavenging, because they were unable to do so by means of predation. It also is worth mentioning that birds and pterosaurs were almost completely absent from the Morrison Formation, so aerial scavengers were not competitors in the environment. While it is possible that preservational biases obfuscate the true abundance of aerial species, we find it unlikely to be true in this circumstance. The Morrison Formation is one of the most well-sampled stratigraphic units in paleontology. It preserves many delicate elements from invertebrates, small mammals, crocodyliforms, turtles, frogs, fish, and even salamanders. Microsites in the Morrison Formation are rich enough that some analyses suggest depositional conditions may have weakly favored preservation of small-bodied and fragile elements. In spite of this, the Morrison Formation preserves virtually no pterosaurs [60]. But even if we assume that pterosaurs were widespread despite this, there is even less evidence to suggest that they were significant consumers of dinosaur carrion. Their shed teeth are not reported from death site quarries; in contrast, theropod teeth are common, suggesting pterosaurs probably were not feeding in those locations. Birds of the time period were crow-sized, so if they existed in the Morrison, they probably were physically unable to process large dinosaur carcasses. It would be challenging to argue that an *Archaeopteryx* relative could have removed hide, muscle, ligamentous tissue, or even soft organs of multi-tonne dinosaur meat, because they simply would not have been strong enough. The most logical explanation is that large theropods were the primary scavengers in the Morrison Formation community, and aerial species—if present—relied on other resources. If researchers discover fossil evidence of vertebrate flying scavengers from the Morrison in the future, our position on this subject will naturally change. But at the time of this writing, almost all data indicates that *Allosaurus* was the most abundant carnivore in the environment, so it is not clear what other species would have been able to appreciably consume sauropod-falls, and we find it unrealistic to assume that this resource pool would have been rare, or ignored. If the authors of [73] had used more realistic assumptions about theropod carcass detection range, and had modeled carrion conditions to include sauropods, we are confident their results would have corroborated our own.

A final distinction between the approach here and in [3] from that of [73] is that the agent-based modeling approach forms part of a class of dynamic models to study interactions among the agents and objects constituting the system. As such, because the outcome relies on interactions among agents with variable characters leading to variable responses, this class of models essentially are stochastic, and are constantly self-adjusting depending on circumstances represented in the virtual sandbox, and this allows them to simulate evolutionary mechanisms. In contrast, the class of static models employed by [73] are not able to answer questions about reproductive fitness potential or heredity.

### Phenotypes

The tailfat attribute was the most important determinant of Allosaur Agent fitness, even more than carcass detection_range, which we expected to be the most critical to foraging success based on our previous modelling outcomes [3]. Allosaur Agents in our study had a significant reproductive advantage if they were able to consume and store more energy than their peers; we therefore hypothesize that in life, their tails were the main fat storage structures for this purpose. It is likely that long, voluminous tails were retained throughout non-avian archosaur evolution partly because of the benefit provided by increased fat storage capacities, and our research reflects this.

The hearing attribute had no relationship to reproductive success of Allosaur Agents; while *Allosaurus* had a poor sense of hearing in life and likely were unable to detect subtle sounds [80], it is possible that the hearing attribute as implemented in the present study was too crude to be meaningful and may not have been a good representation of this adaptation. The same also could be true for the binocular_vision attribute. Binocular vision usually evolves to improve targeting in 3-dimensional space, whether for hand-eye coordination, biting, or to chase down prey, which requires spatial dimensions that this model was not able to reproduce well. Furthermore, as noted above, most large predators engage their prey from much shorter distances than we modeled. However, the fact that binocular_vision partially predicted reproductive success here was surprising, given that killing Prey Agents was not reproductively advantageous. It is not clear why Prey Agent detection was beneficial to Allosaur Agents when killing them was detrimental, and we hypothesize that this result was even more unexpected because in life, large theropods in sauropod-dominated systems had underdeveloped stereoscopic abilities [70]. Additional research may help to understand the evolution of dinosaur vision systems more clearly and hopefully reconcile this discrepancy. Traits such as dominance, carcass detection_range, and tailfat, were much easier to model because they rely on patterns that are easier to describe numerically. For example, because bite_force was based directly on hunting success probability in living predators, we consider it a more representational abstraction than the other two predator attributes.

### Vacant niches

Many ecological roles go unfilled even when resources are abundant and competition absent [81–84]. These vacant niches are even present in mature, diverse marine ecosystems, where sometimes fewer than half of available resource pools are used [85–87]. There are two principal reasons for this absence. First, species are not always optimally adapted for their environments, which is why instances of competitive overlap are uncommon even in “saturated” ecosystems [88]. Second, an important but overlooked aspect of foraging ecology is that resource accessibility often drives consumer-resource decisions. Depending on environmental conditions and functional traits, some energy sources may be nearly impossible for certain animals to exploit if they are inconvenient to capture [81]. This is why wolves avoid hunting bison in favor of elk, even when bison outnumber elk by a factor of 2:1 [88]. Bison are much larger and much more dangerous than elk, which makes elk more evolutionarily advantageous to exploit even when there are fewer of them available. Indeed, as densities of bison increase, wolves more often prefer to scavenge these, rather than targeting less convenient prey of any kind [88]. Alaskan wolves, unfamiliar with bison, do the same thing and assume the role of apex scavenger when winter conditions produce higher densities of ungulate carrion, because carrion is less physiologically expensive to acquire [21]. We hypothesize that this is partially why Prey Agents in our research were less valuable from a fitness perspective than sauropod Carcass Objects, even though Prey Agents posed no survival risk to Allosaur Agents and simply were less convenient to engage.

The concept of vacant niches also applies to examples across long evolutionary intervals. Many sauropods evolved as high browsers to forage among tall conifers, sometimes more than 10 m above ground level [89]. This is important to our study for two reasons. Sauropods have been hypothesized to have evolved long necks so as to extend their feeding envelopes, which thereby allowed them to consume more tree-matter while remaining stationary [90, 91]. According to this hypothesis, they could have spent several hours standing at the base of a conifer, stripping its branches, before moving to an adjacent tree to repeat the process, without expending much energy on locomotion. It is unknown whether this adaptation was a response to mobility limitations imposed by their large size, or if it caused their large size to evolve based on metabolic efficiency gains. However, is unlikely that sauropods could have developed a feeding mode that allowed them to stay in one place for extended periods if they were under constant predatory pressure during a majority of their lives. Prey animals typically abandon feeding opportunities to avoid the risk of predator exposure [92, 93]. Sauropods were slow, and were incapable of doing this: if they felt threatened, they would have been unable to flee quickly even as adolescents, so their survival probably was not dependent on their ability to avoid predators, except as hatchlings and young juveniles. Instead, the ability to exploit a stationary feeding mode suggests that sauropods were able to feed undisturbed for most of their lives, and this would be difficult to explain if the environment was saturated with hunters that were able to disperse them. Sauropod-falls during the dry season likely provided enough calories such that theropods were not forced to hunt as often as today’s large prey specialists, which potentially explains how the two groups of dinosaurs were linked evolutionarily. It is possible that, broadly speaking, sauropod gigantism, via natural-attrition carrion, rewarded the most efficient scavengers, and in doing so, relaxed predation pressure on living sauropods which freed them to evolve progressively increasing size as semi-stationary browsers, in a feedback loop that perpetuated each lineage convergently.

This paleontological scenario contrasts sharply with modern mammalian ecosystems. Mammalian herbivores have not evolved to exploit conifer treetops, not even large, long-necked outliers such as *Paraceratherium* (Mammalia: Perissodactyla: †Paraceratheriidae), which probably consumed mid-level leafy plants [94]. Mammalian herbivore evolution has so far been tied very closely to the emergence of grasses [94–99], which were not widespread in any Mesozoic biome, but became dominant after the K-Pg extinction. Grasses are more convenient resources than conifer treetops because they regenerate rapidly, and—more importantly—are not difficult to access for most quadrupeds. The highly convenient nature of grasses often allows grassland habitats to accommodate the energy needs of multiple species with minimal competitive exclusion [99]. Mammalian large herbivore behavior is also influenced by predation risk, and most have evolved some degree of flight response. Because of this, there has never been enough consistent pressure on herbivorous mammals to force them into high-browse resource pools, nor has there been an opportunity for them to evolve a semi-stationary lifestyle, which has left the high-browser niche completely empty for millions of years. Although research has suggested that land surface area and climate temperature constrained the upper size limit of mammals since the K-Pg extinction [100], we hypothesize that resource pool accessibility conditions strongly contributed to the evolutionary framework that has prevented sauropod analogues from evolving among mammals. Without sauropod-sized large herbivores, carrion resource pools diminished to a point of much lower convenience and profitability. This, in turn, may explain why mammalian analogues of carnosaurian bulk scavengers also have not appeared since the Mesozoic: the absence of sauropod-sized carrion influx forced carnivores to meet their energy budgets by exploiting less convenient resource pools, as also happened with tyrannosaurs.

The apex predator niche is just as likely to be vacant as any other if conditions allow meat resources to be available more conveniently than by hunting, which often is dangerous and always is metabolically expensive [101, 102]. There is direct evidence of apex predator-free environments as well, and one would be remiss not to remark on them again in the context of Komodo dragon ecology, given that they are iconic reptilian carnivores with some similarities to theropods [3, 103]. Komodo dragons are generalist scavengers to such a degree that they evoke no fear response in local herbivores, not even in their putatively preferred prey targets [104]. Because predation risk is so low, deer in Komodo National Park are more frightened of domestic goats than they are of dragons. Ungulate population density in dragon territory is not controlled by predation at all, but by habitat and food availability [104–106]. The apex predator role in Komodo National Park is at least partially vacant, yet scavenging resources and low metabolic costs allow Komodo dragons to exist at high biomass densities when compared to mammalian carnivores [105]. Because *Allosaurus*, and many other theropods in sauropod-dominated systems, also were highly abundant compared to modern mammalian carnivores [12], and were exposed to an overabundance of sauropod carrion meat resources, it is likely that the apex predator niche was ecologically vacant for them as well.

Herbivore density varies with environmental conditions, but dinosaur ecosystems probably supported much more herbivore biomass than any of today’s environments [77], so we should expect that they produced more carrion as well. Average herbivore biomass in the Serengeti, inclusive of all herbivores over 20 kg in adult mass, is estimated at a mean of 6,472 kg per square km, while some European environments reach 16,000 kg/ sq km [107]. Mammoth steppe biomes likely were similar, with potentially 10,500 kg per sq km [108]. Dinosaur productivity estimates have so far been much higher. Authors have estimated it to be between 186,000 to 672,000 kg per square km in Late Jurassic due to the presence of sauropods [56]. Other researchers have hypothesized that global Late Jurassic terrestrial herbivore biomass may have been up to 22.3 times higher than today’s mammalian biomass if dinosaurs were endotherms, or 67.29 times higher if ectotherms [77]. An ecosystem with 10 or 15 sauropods per square km would have necessarily generated more carrion each year than any modern biome. Especially in the Morrison Formation, where dinosaurs inhabited a harsh environment and experienced severe drought-induced mortality events each year [109, 110]. We will never know the true population densities of dinosaurs in the Late Jurassic, but based on the evidence we have, we are confident that environmentally-induced sauropod-falls were common in the Morrison Formation and carrion resources would not have been difficult for theropods to rely on. In addition, we reiterate that the Morrison preserves very few pterosaurs, and virtually no avian taxa [60] so aerial scavengers probably were not competitively significant to allosaurs if they even were present at all. This itself would be a significant anomaly if allosauroid dinosaurs were not bulk scavengers, because it would mean scavenger niche resource pools were unused in a time when terrestrial carrion abundance was orders of magnitude more available—and less energetically expensive to access—than it is today.

Many factors contribute to foraging ecology in life, but broadly speaking, there may be an inflection point where a resource pool is abundant enough to be profitable, but not convenient enough to exploit, or conversely, highly convenient but not profitable. Our results thus demonstrate that meat-eating dinosaurs that lived alongside sauropods likely evolved to get most of their calories by scavenging, and while they certainly hunted for prey occasionally, as almost all carnivores do, they would not have had to rely on predation to survive because their primary resource pool was both highly profitable and highly convenient. The existence of sauropod carrion as a resource almost certainly caused the apex predator niche to be at least partially vacant, not just in the Morrison Formation, but in most other sauropod environments as well. It is worth mentioning that sauropods probably were among the most inconvenient terrestrial prey targets ever to evolve due to their characteristically great size [4] and consequent power. But their carcasses probably were among the most convenient terrestrial meat resources ever to appear [3].

### Resource use and life history

Dinosaurs experienced ontogenetic shifts in foraging ecology [111]. Modern animals also use different resource pools as they progress to adulthood, and comparison among them allows us to detail this phenomenon analytically. Young and adolescent great white sharks, for example, are effective generalists that focus on fish and other small prey. As they age and their diets shift to marine mammals, bite force increases dramatically to meet the needs of macropredation [112]. But in mid- to late-adulthood, when sharks reach a particular mass, they lose substantial speed and maneuverability. It then becomes difficult for them to target smaller, faster seals, at which point they become primarily scavengers of whale carrion. While they continue to hunt throughout their lives, research suggests that they do so much less in maturity because it is more profitable, and much more convenient, to search for dead and dying whales, rather than to engage in the tests of agility required to hunt pinnipeds. In fact, they appear to quit hunting when whale calories are available [112–114]. Similarly, Komodo dragon young are agile climbing predators of insects and small vertebrates, whereas—as noted above—adults are scavengers or opportunistic cannibals on their own young [115, 116], which is one reason why they are not analogues of mammalian apex predators.

Carnosaurs such as *Allosaurus* probably were like sharks in some respects. They likely began life as opportunistic generalists that concentrated on invertebrates and smaller vertebrate prey. But unlike sharks, we hypothesize that they may have skipped the macropredator life history phase altogether, moving straight into the bulk scavenging phase during adolescence. This interpretation is supported by our computational results, and also agrees with other research on dinosaur foraging behavior. For example, tyrannosaurids transitioned between different foraging strategies as they matured, beginning life with weak jaws and laterally compressed, knife-blade teeth. During adolescence, these morphological attributes transitioned into jaws with high bite force and sturdy, incrassate dentition, hypothesized to overcome stresses associated with predation on megaherbivores [117]. This fact strongly suggests that ziphodont dentition alone was inadequate for megaherbivore predation in dinosaurs. Otherwise, tyrannosaurids would not have experienced selective pressure to favor this expensive adolescent transformation. They were forced to abandon it in favor of powerful jaws to successfully hunt other dinosaurs because tyrannosaurids existed largely without the benefit of sauropod carrion in their ecosystems. This also follows logically, because cranial rigidity and high bite force evolve in predators so they can overcome physical stresses required to subdue and kill struggling prey [118].

On the other hand, it is known that *Allosaurus*, and most other theropods that lived alongside sauropods, maintained weak, flexible jaws and ziphodont dentition throughout their entire lives [119, 120]. This in and of itself is strong evidence that, unlike great white sharks and tyrannosaurs, *Allosaurus* did not undergo a dietary shift toward large herbivore predation as they approached maturity. It is probably unrealistic to characterize allosaurs as specialized bloodletters with unique, weak-jawed hunting styles, either, because there is no evidence to support such an explanation. Even canids, which often kill prey by exsanguination, have very high bite force quotients along with morphologically slender canine teeth to successfully accomplish the soft tissue damage required to bleed out their targets [121, 122]. Canids also have excellent binocular vision, which allows them to make precise bites into arteries and other vulnerable anatomy of their prey. *Allosaurus* and its carnosaurian relatives uniformly lacked the high bite force, dental adaptations, and binocular vision, that would have allowed them to consistently undertake these types of attacks. Data from our model corroborates this and provides a possible causation mechanism as well, because Allosaur Agents did not experience pressure to evolve attributes that increased hunting proficiency, despite being exposed to unlimited Prey Agents. Instead, they evolved to exploit more convenient sauropod carcasses, even though our model almost certainly underestimated the true quantity of sauropod carrion produced on the landscape during the Age of Giants. Allosaur Agents were most likely to reproduce when they had high meat consumption and high fat storage, along with extended carcass detection range, which are among the many other adaptations they share convergently with modern vultures [3]. As we previously demonstrated, sauropod carrion was perhaps even more available to dinosaurs than today’s whale carcasses, because whale carcasses typically float over long distances before they either wash ashore or sink into benthic communities. In life, sauropod cadavers were not subject to such factors of unpredictability.

## Conclusions

It is worth emphasizing that modeling projects are meant to represent oversimplified abstractions of complex systems. They are not meant to perfectly replicate natural conditions, yet, even as abstractions, models of this type often produce useful, coherent data about systems that are difficult or impossible to directly observe, which is essential for constructing valid theories. In the present instance, this loss of modeled variables could have made our arguments either much stronger, or much weaker. Had we modeled evolutionarily mature populations of multiple sauropod species, which we know were present in the Morrison Formation, the resulting carrion abundance would have made other resource pools less desirable no matter how we modeled them. We also could have modeled multiple species of competing predatory Agents rather than Allosaur Agents only, perhaps with males and females, breeding seasons, nesting and gestational intervals, pack hunting, territorial competition, age classes, or many other known factors of animal life histories. These factors all certainly would have affected how theropods prioritized resource use as well, potentially to favor predatory specialization under particular circumstances. If hearing and binocular_vision had been programmed differently, perhaps in a model with greater realistic depth, these factors also might have influenced the success of Allosaur Agents as predators. The purpose of our research was to understand how carrion availability in a world of sauropods would have influenced consumption patterns and adaptations of carnivores. In this case, we have been able to describe theropod foraging ecology with fewer assumptions than other paradigms. It does appear that large theropods may not have evolved as analogues of today’s apex predators, but instead, obtained most of their calories from megaherbivore carrion. Our data analysis suggests that selective pressure on dinosaurs likely favored this foraging regime very strongly, and allowed us to demonstrate strong support for the hypothesis that sauropod carrion was a primary driver in the evolution of feeding ecology in carnosaurian dinosaurs.
